# Nematodes enhance plant growth and nutrient uptake under C and N-rich conditions

**DOI:** 10.1038/srep32862

**Published:** 2016-09-08

**Authors:** Mesfin T. Gebremikael, Hanne Steel, David Buchan, Wim Bert, Stefaan De Neve

**Affiliations:** 1 Department of Soil Management, University of Ghent, Ghent, Belgium; 2 Department of Biology, Nematology Research Unit, University of Ghent, Ghent, Belgium

## Abstract

The role of soil fauna in crucial ecosystem services such as nutrient cycling remains poorly quantified, mainly because of the overly reductionistic approach adopted in most experimental studies. Given that increasing nitrogen inputs in various ecosystems influence the structure and functioning of soil microbes and the activity of fauna, we aimed to quantify the role of the entire soil nematode community in nutrient mineralization in an experimental set-up emulating nutrient-rich field conditions and accounting for crucial interactions amongst the soil microbial communities and plants. To this end, we reconstructed a complex soil foodweb in mesocosms that comprised largely undisturbed native microflora and the entire nematode community added into defaunated soil, planted with *Lolium perenne* as a model plant, and amended with fresh grass-clover residues. We determined N and P availability and plant uptake, plant biomass and abundance and structure of the microbial and nematode communities during a three-month incubation. The presence of nematodes significantly increased plant biomass production (+9%), net N (+25%) and net P (+23%) availability compared to their absence, demonstrating that nematodes link below- and above-ground processes, primarily through increasing nutrient availability. The experimental set-up presented allows to realistically quantify the crucial ecosystem services provided by the soil biota.

It is well documented that nitrogen (N) inputs into the soil significantly influence aboveground ecosystem productivity and below-ground pools and fluxes of N such as microbial biomass N and mineralization^1^,^2^. Despite decades of intensive research, it remains difficult to quantify and predict such crucial processes in natural or man-made ecosystems^3^. The role of soil fauna, in particular, remains far from being elucidated, primarily because of a lack of quantitative data gathered from realistic soil environments^4^. Nematodes are among the most abundant and diverse soil fauna that link below- and above-ground processes such as soil organic matter (SOM) decomposition and plant production^5^,^6^,^7^. The ecological significance of nematodes in these processes is attributed to their highly diverse feeding behaviors that have enabled them to adapt to virtually every habitat^8^ and to the key positions they occupy in the below-ground food web^9^. For instance, plant-feeding nematodes are known to increase the release of carbon-rich root exudates into the soil, thus stimulating microbial growth^10^,^11^ that probably leads to an increased SOM decomposition through a mechanism known as a ‘priming effect’^12^,^13^. Bacterivorous, fungivorous and omnivorous nematodes feed on these microbes and excrete nutrients in excess of their metabolic need, primarily as plant available organic and inorganic forms such as amino acids, NH_4_^+^ and PO_4_^−3^ 14, increasing plant uptake, in a similar way as in ‘microbial loop’ mechanism^15^,^16^. The abundance and activity of these microbivorous nematodes may, in turn, also be regulated by predatory nematodes and other fauna, further modulating nutrient availability^17^,^18^.

Despite such strong interactions between functionally diverse groups of nematodes, microbes, and plants, most of the experimental findings on the roles of nematodes in below-ground processes are often based on highly artificial experiments with only a few species of selected nematode functional groups and their respective prey(s), and often in the absence of plants^19^,^20^,^21^. Such studies have been used in soil food web modeling and have increased our understanding of the roles of nematodes in the process of nutrient mineralization and plant uptake^22^. However, the findings obviously do not account for the collective role of nematodes and are far from simulating the reality in the field. Data from realistic laboratory experiments that emulate field conditions much more closely are an absolute necessity for improving ecosystem models. Yet, studies in the presence of plants are few and have so far led to inconsistent results^14^,^23^,^24^.

The recent understanding of SOM decomposition and nutrient mineralization processes is that available C and N not only regulate the mineralization processes mediated by microbes^3^,^25^, but also influence the contribution made by soil fauna^4^. In line with the ‘N mining’ theory, which hypothesizes that low N availability enhances microbial degradation of soil organic matter^26^, it has also been suggested that the contribution of nematodes and other soil fauna becomes significant usually under conditions of low nutrient availability^14^,^27^,^28^.

Given that many ecosystems globally are experiencing increased inputs of anthropogenically derived N^2^,^29^, it is important to understand to what extent this may affect the functionally diversified groups of nematodes and their role in nutrient availability and plant production. Previous studies have shown how the structure and functional capabilities of soil microbial communities respond to N additions^2^. So far, there are no data on whether and how the entire nematode community contributes to nutrient mineralization under nutrient-rich conditions, particularly N. Such data are crucial to enhancing our understanding of how soil fauna respond to increasing N availability for microbes and plants in agricultural ecosystems and what the feedbacks to plant productivity are.

In this study, grass-clover with a low C:N ratio was applied as an external source of C and N to create C and N-rich conditions. We hypothesized that increased rhizodeposition due to below-ground herbivory by plant-feeding nematodes, in combination with C and N from a nutrient-rich organic amendment would boost microbial growth and activity. This, in turn, would attract microbivorous nematodes, particularly enrichment opportunist bacterivores and general opportunist fungivores, resulting in an increased release of plant-available nutrients and a concomitant increase in plant biomass production. Previous ecological studies on the role of nematodes in nutrient cycling suffered from oversimplification of the setups. Here, we tested this hypothesis using a novel experimental setup that mimics field conditions regarding structure and abundance of the native microbial and nematode communities, the application of organic inputs, the presence of plants and soil. The results demonstrate how nematodes link below- and above-ground processes primarily through acting on nutrient availability.

## Results

### Nematode abundance and community structure

**Figure 1:**
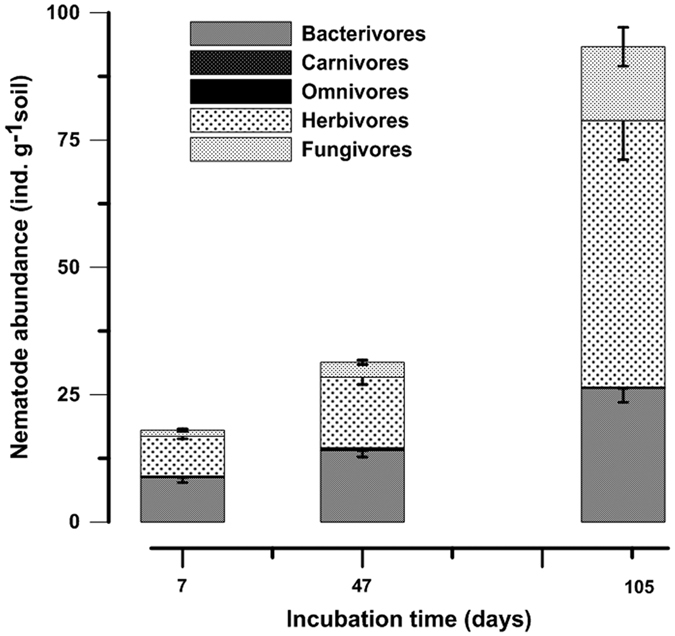
Mean nematode abundance of each trophic group in reinoculated samples (+Nem) on the 7th, 47th and 105th days after incubation. The −Nem treatment is not presented as this treatment was without nematodes. The error bars indicate the standard error of the mean (n = 3).

The abundance and functional composition of nematodes in reinoculated samples (+Nem) was similar (18.0 vs. 18.3 ind. g^−1^ dry soil) to the non-irradiated fresh control samples (CTR). All the nematode taxa identified in the CTR were also identified in the reinoculated samples and showed no significant differences in abundance of each trophic group (S1). No significant differences were found for any nematode indices between the CTR and +Nem treatments at the beginning of the experiment (S2). The total abundance and the abundances of herbivores, bacterivores and fungivores significantly increased (p < 0.01) over time in reinoculated samples (Fig. 1). The bacterivores significantly increased (p < 0.05) from day 7 to day 47 and from day 47 to day 105, while fungivores and herbivores increased only from day 47 to day 105. The trophic composition was dominated by both herbivores (43%) and bacterivores (47%) at the beginning of the incubation. However, by the end of the incubation herbivores dominated (55%) over bacterivores (28%) and fungivores (15%), even though the latter two groups significantly increased in abundance over time (Fig. 1).

### Microbial biomass C and community structure

**Figure 2:**
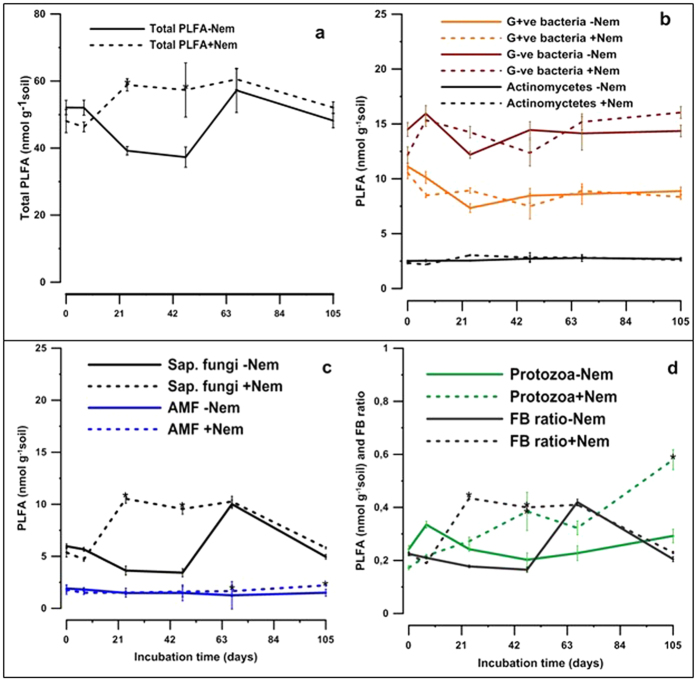
The evolution of total PLFA (**a**) and PLFA biomarker concentrations of the major microbial groups G+ve, G−ve bacteria and actinomycetes (**b**), Saprophytic fungi and AMF (**c**) and protozoa nad FB ratio (**d**) over time in +Nem and −Nem treatments. The asterisk symbol (*) indicates statistically significant differences between treatments on the corresponding sampling dates.

The mean microbial biomass C (C_mic_) in the +Nem treatment was not significantly different (p = 0.178) from −Nem (Table 1). Total PLFA concentration tended to be higher in +Nem treatments than −Nem treatments during most of the incubation period with significant differences on day 24 (+19.65 nmol g^−1^ dry soil, p = 0.025) and 47 (+20.04 nmol g^−1^ dry soil, p = 0.021) (Fig. 2a). No significant interactions were observed between incubation time and nematode treatments for bacterial and actinomycetes communities (Table S4). No significant differences were found in average abundances over time, between +Nem and −Nem treatments for marker PLFAs of Gram-positive (p = 0.959), Gram-negative (p = 0.337) and actinomycetes (p = 0.784). Saprophytic fungi marker PLFA concentrations were significantly higher in +Nem than −Nem on day 24 (+6.90 nmol g^−1^soil, p = 0.000) and 47 (+6.12 nmol g^−1^ soil, p = 0.000) and AMF biomarkers on day 67 (+0.40 nmol g^−1^ soil, p = 0.002) and on day 105 (+0.71 nmol g^−1^ soil, p = 0.000) (Fig. 2c). As for the fungal marker PLFAs, the F:B ratio was significantly higher in +Nem than −Nem treatment on day 24 and day 47. Protozoa marker PLFA concentrations were significantly higher in +Nem than −Nem treatments on day 47 (+0.18 nmol g^−1^ soil, p = 0.004) and on the last day of incubation (+0.28 nmol g^−1^ soil, p = 0.000) (Fig. 2d).

### Total plant dry biomass and nutrient uptake

**Figure 3:**
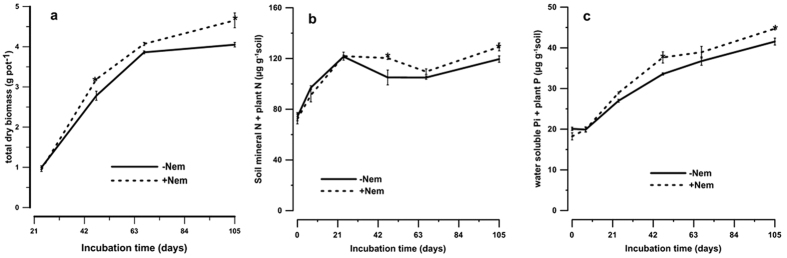
The evolution of dry biomass which is the sum of both the shoot and root biomass (**a**), and total nutrient mineralization (sum of available nutrients in the soil and plant uptake at each sampling occasion) for Nitrogen (μg N g^−1^ soil), (**b**) and Phosphorus (μg P g^−1^ soil), (**c**) in +Nem and −Nem treatments. The error bars indicate the standard error of the mean (n = 4).

Total dry biomass (root and shoot dry weight) was higher in +Nem than −Nem samples throughout the incubation (Fig. 3a) with significantly higher differences on day 47 (+21%, p = 0.002) and day 105 (+15%, p = 0.000). Nitrogen and phosphorus uptake was significantly higher in +Nem than in −Nem treatment with overall mean differences of +6% (p = 0.003) and +10% (p = 0.002), respectively (Table 1).

### N and P dynamics

NH_4_^+^-N and NO_3_^−^-N concentrations in the soil were not significantly different between the treatments (Table S4). However, the total available N (the sum of mineral N in the soil and N taken up by the plants), was significantly higher in +Nem than in −Nem on day 47 (+14%, p = 0.002) and day 105 (+8%, p = 0.042) (Fig. 3b). The net N mineralized, which was calculated as the difference in total available N at the start (day 0) and end of the incubation was 56.8 and 45.4 mg N kg^−1^soil in +Nem and Nem, respectively, i.e. about 25% higher in +Nem.

As was the case for mineral N, water soluble inorganic P (P_i_) concentration in the soil was not significantly different (p = 0.874) between the treatments (Table S4). However, the total available P (the sum of inorganic P in the soil and P taken up by the plants), was significantly higher in +Nem than in −Nem treatments on day 47 (+12%, p = 0.001) and final sampling time (+8%, p = 0.008) (Fig. 3c). The increase in available P during the incubation, i.e. the difference in total available P at the start (day 0) and end of the incubation, was 26.5 and 21.4 mg N kg^−1^ soil in +Nem and −Nem, respectively, i.e. about 23% higher in the +Nem treatment.

## Discussion

Reconstructing soil foodwebs have been widely employed by soil ecologists to investigate soil ecological processes^5^,^19^. If soil sterilization is used, quantitative extraction and reinoculation of microorganisms are crucial to reconstruct soil foodwebs successfully. However, quantitative extraction of representative microbial communities is challenging as they are strongly attached to the soil particles^30^. Moreover, the spatial distribution of the reinoculated microbial communities is different from the indigenous ones^31^ which may lead to overgrazing by the reinoculated microfauna, and result in an overestimation of their roles^21^. The possibility of leaving a representative native microbial community by the application of low doses of gamma irradiation, thus avoiding the need for extraction and reinoculation, has recently been shown in previous studies^32^,^33^ in which the microbial properties of irradiated fresh soil were compared with unirradiated control soil. Similarly, in the current study, the total PLFA concentration and the PLFA biomarker concentrations of the major microbial groups such as Gram-positive, Gram-negative bacteria, actinomycetes, saprophytic fungi, AMF and protozoa in +Nem samples were comparable to those in unirradiated CTR samples at the beginning of the incubation experiment (Table S3). The total PLFA concentration found in this study, which is an indication of active microbial biomass, was also comparable to the concentration reported in similar organically amended fresh soils^34^, further confirming the representativeness of the microbial community left in the irradiated treatments.

Nematode reinoculation was also very successful both regarding abundance and diversity in comparison with the non-irradiated control soil (CTR) as shown in Table S1 and S2. All the taxa that were identified in the CTR were also identified in the +Nem samples. Moreover, all the nematode indices of the +Nem samples were comparable to those of the CTR samples showing the simulated mesocosms were representative of field conditions regarding nematode abundance and community structure. As the objective of the experiment was to study the collective contribution of all functional groups of nematodes, the presence of bacterivores, fungivores, carnivores, omnivores and herbivores with a high degree of resemblance to field conditions (CTR) was an essential prerequisite.

In previous studies, only selected species of bacterivorous nematodes were found to increase plant N and P uptake significantly^14^,^35^. When the entire nematode community was considered in soils without additional OM, the effect on N uptake^23^,^24^ was not significant. Here, in line with our hypothesis, interactions between nematodes, microbes and plants increased net N (+25%) and net P (+23%) availability and subsequent plant growth (+9%), demonstrating tangible effects of nematodes on nutrient mobilization under C-and N-rich conditions, which is often the case in agricultural ecosystems where the application of nutrient-rich organic amendments is a common practice.

**Figure 4:**
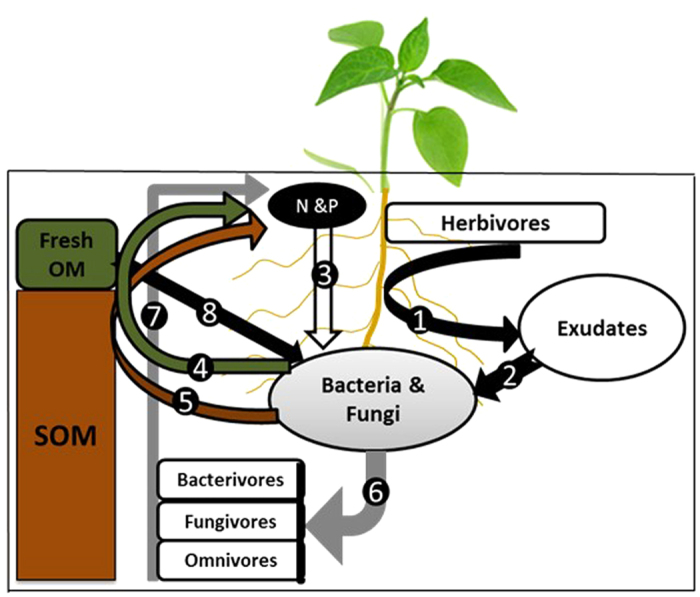
The main mechanisms by which nematode functional groups link below- and above-ground processes under resource-rich conditions. Root herbivory (1) increases root exudation stimulating bacterial and fungal growth (2). Microbial communities then immobilize (3) N and P from the bio-available pool and N and P acquired from that of the externally added fresh OM (4) and the native SOM (5) through a mechanism known as root priming effect. Bacterivores and fungivores feed on the microbial communities (6) and excrete N and P in excess of their metabolic needs through a mechanism known as microbial loop (7) which otherwise would have been locked up in the microbial biomass. By increasing available nutrients, nematodes enhance plant nutrient availability and uptake. Depending on the chemical composition, the fresh OM would stimulate the microbial biomass and activity (8) in a way similar to that of exudates.

Figure 4 illustrates two possible primary mechanisms explaining how these multitrophic interactions and the application of external grass-clover influence nutrient availability and plant growth. Firstly, rapid microbial growth is stimulated (as shown by significantly higher PLFA concentration in +Nem treatment, Fig. 2a), possibly as a result of increased C-rich root exudation due to root herbivory and labile C from the fresh grass-clover. A small infestation of plant roots by herbivorous nematodes increases ‘leakage’ of rhizodeposits that boost the microbial growth as shown by several authors^5^,^10^,^11^,^36^. The trophic composition was significantly shifted in favor of herbivores gradually during the incubation (Fig. 1), further indicating the role of herbivory in stimulating microbial growth.

Nematodes, particularly bacterivores have also been shown to change the composition of the microbial communities, particularly the bacterial community^21^,^37^,^38^. In contrast to these previous studies that reported shifts in bacterial community structure based on a few selected species of nematodes, the presence of the entire nematode community did not change the abundance of the major bacterial groups in the current study (Fig. 2b). On the other hand, the presence of the entire nematode community temporarily changed the abundance of the saprophytic fungal and AMF communities. The abundance of the AMF PLFA biomarker was significantly (p < 0.05) correlated with herbivores (r = 0.81), bacterivores (r = 0.9) and fungivores (r = 0.79), suggesting that AMF were positively influenced by interactions between plants and different functional groups of nematodes. The AMF may also have benefitted from the presence of different functional groups of nematodes possibly through increased N availability^39^ and improved root architecture^40^ that might have enhanced the N nutrition of AMF^41^ and eventually increased its biomass. AMF have also been shown to induce systemic resistance against plant parasitic nematodes and other pathogens, which enhance plant growth and rewards the plant for supplying C to the AMF^42^. Previous studies with similar experimental setups, but in the absence of plants reported a higher fungal abundance in clover-amended microcosms in the presence of nematodes^43^.

Secondly, when microbivorous nematodes graze on the soil microflora, they excrete nutrients in excess of their metabolic needs in the form of NH_4_^+^, PO_4_^−3^ and low molecular weight compounds such as amino acids^9^,^14^. In the current study, enrichment opportunists nematodes (cp1 and cp2) that feed on bacteria and fungi, respectively, dominated the nematode community following the addition of fresh grass-clover as also reported in previous studies e.g. Ferris and Bongers^44^. Such a rapid growth of microbivorous nematodes may have likely resulted in a higher N and P release probably in a similar way as increase in population size of bacterivores increased C mineralization^45^. The presence of high and significant correlation coefficients between bacterivores and both N (r = 0.67, p < 0.05) and P (r = 0.93, p < 0.01) uptake, and the significant increase in abundances of bacterivorous and fungivorous nematodes over time (Fig. 1) further support this hypothesis.

The influence of nematodes on P mineralization and plant P uptake have been poorly investigated^46^, partly because P release is thought to be controlled more chemically than microbially^47^. However, it has been acknowledged that microbes are actively involved in mobilizing soil P^48^. In previous studies, bacterivorous nematodes were also shown to contribute to P availability and plant P uptake^49^. This is the first study that quantified the collective contribution of different functional groups of soil nematode communities to P mineralization. Although the mechanisms explaining this still need to be investigated, findings from the present and previous studies suggest the importance of the excretion of excess P by microbivorous nematodes and stimulation of AMF fungi which have been shown to increase both N and P uptake^50^.

Interactions between protozoa and nematodes in +Nem treatments might have also enhanced N and P availability^51^,^52^. At the beginning of the incubation both treatments had comparable protozoa communities (based on PLFA biomarkers) that survived the irradiation process. However, during the incubation period, PLFA biomarkers of protozoa significantly increased in +Nem treatments compared to −Nem suggesting increased grazing activity as a result of interactions between protozoa and nematodes. Plants compete for nutrients that become available by the grazing effect of these nematodes and protozoa that otherwise would be immobilized by microbes, as suggested by the ‘microbial loop’ model^15^,^16^. However, biomarker PLFAs for protozoa are not very specific, and therefore, the possibility of a higher abundance of protozoa in +Nem would need further confirmation from additional analysis.

The nutrients released through nematode grazing may become immobilized and/or taken up by the plants depending on various factors such as C:N:P ratio of the substrate^46^,^53^. Based on the low C:P ratio of the soil (13.3:1) and low C:N ratio of the substrates in the current experiment (Grass-clover = 10.1:1, soil before amendment = 12.3:1), N may become available for the plant uptake as microbial immobilization is expected to be low^54^. On the other hand, extra C-rich root exudation due to root herbivory may contribute for considerable microbial immobilization. Therefore, depending on how much extra C became available due to herbivory, the microorganisms might have decomposed the native SOM to obtain additional N for their metabolic need according to ‘priming effect’ mechanism. This hypothesis is further supported by the presence of significantly higher N and P mineralization in the presence of nematodes during most of the incubation time (Fig. 3b,c). The increase in dry biomass yield is also related to increased plant nutrient uptake as shown by the significant correlation between dry biomass yield and N uptake (r = 0.69, P < 0.05) and P uptake (r = 0.93, p = 0.01). Our data suggests that plants may have outcompeted the microorganisms for the available nutrients. Under conditions of low nutrient availability, roots may lose the competition with microbes for nutrients whereas at higher N availability (with low C:N ratio substrates) and longer term experiments such as in the current study, the competition between microbes and roots for N would be less and possibly outcompeted by root^3^,^54^. Apart from nutritional effects, nematodes have been shown to increase root proliferation as a result of grazing-induced plant hormone production, which may also be responsible for such increase in plant biomass^55^,^56^. An increase in shoot biomass, particularly by the presence of selected species of bacterivorous nematodes, has been recorded in different experiments^14^,^35^.

It is generally expected that the bacterial decomposition pathway dominates with substrates of low C:N ratio^57^. Here, the fungal communities and the associated F:B ratio in the presence of nematodes dominated after the first week of the incubation period (Fig. 2c,d). Moreover, the nematode channel index (CI), which indicates the predominant decomposition pathway, significantly increased over time (Table S2), suggesting the dominance of the fungal channel during the decomposition process. Previous studies showed that the decomposition pathway also depends on the availability of N and P in the soil, as the fungal role was found dominating with substrates with low C:N under P-limiting condition^57^. Given the lower C:P ratio of the soil, P-limiting condition does not seem to be the case here, but further data on microbial C:P ratio is required to determine whether microbial P limitation was the case in the current study. Plant parasitic nematodes are also believed to induce the degradation of Cellulose and increase the fungal biomass^10^,^58^ which could be the reason for the dominance of fungal role in the decomposition process in the current study. Given that below-ground ecosystem functions such as SOM decomposition and the associated processes of nutrient transformation are primarily carried out by microorganisms^59^, the impact of nematodes on the growth and turnover of microbial community has important implications for global ecosystem functioning.

In conclusion, while herbivorous nematodes stimulate microbial growth and activity (such as OM decomposition) through increasing root exudation, microbial grazing by microbivorous nematodes increases nutrient availability. Thus, despite the expected yield loss by herbivorous nematodes, at low levels of infestation (as in the current study) and in the presence of the entire free-living nematode community, plants may benefit from the further interactions of herbivores with microbes and microbial grazers, eventually (counter-) balancing the negative effects. This is in agreement with previous studies that reported an increase in the active microbial biomass and nutrient availability in the presence of plant parasitic nematodes at a density (of a single species) ranging from 25 to 69 nematodes per plant^5^,^60^. In the current study, the total abundance of herbivorous nematodes was 93 per plant at the start of incubation and increased to 620 per plant at the end of the incubation. Given the diversity in this herbivorous community, this abundance is still below the threshold for economic damage. The herbivorous community, particularly sedentary endoparasitic nematodes such as *Meloidogyne* have been shown to produce enzymes facilitating organic matter decomposition^58^ as well as increasing N, P, and K concentrations in plant biomass^61^. Our study provides realistic experimental evidence for significant effects of free-living soil nematodes on N and P mineralization under C and N-rich conditions, showing how nematodes link OM decomposition and plant biomass production through increased nutrient availability. Our novel experimental approach that allowed successfully to study interactions between the native microbial and the entire nematode communities can readily be used in other ecological studies to assess the roles of other faunal groups in soil ecosystem functioning. Data on the actual contribution of the entire community of particular groups of soil fauna is crucial to monitor below- and above-ground ecosystem functioning and improve current ecological models which often use inputs from simplified experiments.

## Materials and Methods

### Sample collection

Several augerings were collected in a zigzag pattern to make a composite soil sample from the 0–15 cm layer of an organically managed agricultural experimental field in Merelbeke, Belgium, sown with a grass-clover mix. At the time of sampling the soil was characterized by 1.49 Mg m^−3^ bulk density, 5.2 pH(KCl), 1.23% organic C, 0.10% total N, and 22.3 mg mineral N kg^−1^ dry soil, 256.6 mg P kg^−1^ available P (Ammonium lactate extraction^48^) and 922.6 mg kg^−1^ total P^62^. A bulk sample of fresh grass-clover (C:N ratio 10.1:1) was also collected from the same field, chopped into small pieces and used as an organic amendment in the experiment.

The composite soil sample was gently sieved through a 5 mm mesh to homogenize it and to remove stones and visible soil organisms such as earthworms. Half of the composite soil sample (~20 kg) was kept separately for nematode extraction, the other part (~20 kg) was kept for the incubation experiment following selective sterilization (irradiation) as explained below. The chopped grass-clover was applied at a rate of 4.96 tons DM ha^−1^, i.e. an amount that would be commonly incorporated in the field. This rate is equivalent to 3.5 g DM kg^−1^soil (or 1459 and 137 μg C and N g^−1^ dry soil respectively) taking the surface area, 10 cm depth, and the moisture content of the fresh grass-clover into account.

### Gamma irradiation

For gamma irradiation, seven large sized PVC columns (h = 30 cm, d = 10.8 cm) were each filled with the 3 kg soil mixed with grass-clover at a bulk density of 1.4 Mg m^−3^. By an appropriate choice of irradiation dose, all nematodes and higher soil fauna were killed without significantly altering the microbial communities (bacteria and fungi) as described in detail in Buchan *et al*.^63^ and Gebremikael *et al*.^24^. The PVC columns were subjected to a three kGy dose of gamma irradiation at the Synergy Health sterilizing company, Etten-Leur, The Netherlands. The exact dose applied to each PVC column was measured with dosimeters placed at the top, middle and bottom of each PVC column, the average dosimeter reading in kGy being 3.07 ± 0.34 (mean ± s.d., n = 6).

### Treatments and experimental setup

The experiment had two treatments each with four replicates: 1) defaunated and reinoculated with nematodes (+Nem) and 2) defaunated and without nematodes (−Nem). A total of 48 experimental units was prepared by filling up PVC cores (h = 7.55 cm d = 7.55 cm) each with 400 g of amended and irradiated soil and gently compacted to a bulk density of 1.4 Mg m^−3^. Half of the cores (n = 24) were reinoculated with a concentrated nematode suspension extracted from the same amount of soil (that is, from 400 g soil) by dripping a nematode suspension (about 10 ml) on the soil (using a separate pipette per core), followed by gentle mixing before filing the PVC cores. Each core was sown with 36 seeds of perennial ryegrass (*Lolium perenne*), and 28 seedlings were left after germination. The incubation experiment was carried out for 105 days in a growth chamber at a constant temperature (17 °C) and light regime (16/8 day/night regime). The moisture content of each sample was kept constant at 50% WFPS by adding demineralized water as needed, once a day at the beginning of the experiment and twice a day as evapotranspiration increased following the rapid growth of the grass. Four replicates of each treatment were removed and destructively sampled on day zero and after 7, 24, 47, 67, and 105 days of incubation for chemical (N and P) and biological analysis, namely, phospholipid fatty acid (PLFA), microbial biomass C, nematode abundance and identification. Four replicates of unirradiated fresh soil (CTR) were also destructively removed, but only once (after seven days of incubation) for microbial and nematode analyses as a reference measurement against which the success of the soil foodweb reconstruction was evaluated (detailed protocol for microbial and nematode analysis is provided in the supporting information).

### Mineral nitrogen and phosphorus dynamics

Evolution of mineral N in the soil samples was monitored throughout the incubation by extracting mineral nitrogen (NH_4_^+^-N and NO_3_^−^-N) from 30 g moist soil with 150 ml 1 M KCl (1:5 ratio). Water extractable P was extracted from 2 g dry soil with 20 ml water (1:10 ratio) according to De Bolle, *et al*.^48^. The solution was then filtered through a 0.45 μm membrane filter and inorganic P in the filtrate was determined colorimetrically at 882 nm (Varian Cary 50 spectrophotometer) following procedure by Murphy and Riley^64^. The sum of mineral nutrients extracted from the soil and nutrients taken up by the plant was considered as the total available nutrient concentration (i.e. the sum of what was initially present in the soil and what was mineralized during the experiment).

### Plant nitrogen and phosphorus uptake

Total N in the roots and shoot biomass was separately determined by Elemental analysis (Variomax CNS analyzer, Elementar, Germany) as explained in Gebremikael *et al*.^24^. N uptake was calculated as %N in the plant sample (shoot and root) multiplied by the total dry plant biomass collected at each sampling time and reported as mg N per tube.

Total P in the plant samples was extracted by a combination of ashing and wet digestion with 1N HNO_3_^62^. Total P concentration was determined according to the colorimetric Nitro-vanado-molybdate method of Hogue *et al*.^65^ using a Varian Cary 50 spectrophotometer at 425 nm. P uptake was also calculated in the same way as N uptake, but from the shoot biomass only, as the root biomass was not sufficient for P analysis.

### Nematode extraction and analysis, microbial biomass C and PLFA analysis

The same procedures as in Buchan *et al*.^63^ were followed to extract and analyze the nematode and microbial community structure (Details are available in the supporting information, S1).

### Statistical analysis

Two-way analysis of variance (ANOVA) was applied as the experiment followed a factorial design with two fixed factors: Factor 1) nematodes with two levels: +Nem and −Nem, Factor 2) incubation time with six levels: 0, 7, 24, 47, 67 and 105 days of incubation. The normality and homoscedasticity of the data were checked for all the parameters and log transformation was done for the variables that violated the assumptions. Whenever the interaction between nematodes and time was non-significant (p > 0.05), the main effects were compared, and Fisher’s least significant difference (LSD) was used to analyze mean differences. However, when the interaction term was significant (p < 0.05), a one-way ANOVA model was fitted for each nematode-time combination and Bonferroni’s method was used for the post hoc pairwise mean difference analysis. The two-tailed bivariate correlation was carried out on selected variables. The PLFA data were further analyzed by Principal Component Analysis (PCA) based on the correlation matrix after checking for sampling adequacy using the Kaiser-Mayer-Olkin (KMO) test. All statistical analyses were done using IBM SPSS Statistics 20 software (SPSS inc., Chicago, USA).

## Additional Information

**How to cite this article**: Gebremikael, M. T. *et al*. Nematodes enhance plant growth and nutrient uptake under C and N-rich conditions. *Sci. Rep*. **6**, 32862; doi: 10.1038/srep32862 (2016).

## Supplementary Material

Supplementary Information

## Figures and Tables

**Table 1:** Mean plant uptake of nitrogen and phosphorus and mean microbial biomass C in +Nem and −Nem treatments.

Factor/treatment	Plant nutrient uptake		
	Plant N (mg per pot)	Plant P (mg per pot)	C _mic_ (mg per kg)
Nematode			
minus Nem	38.92(0.73)	7.74(0.50)	490.11(19.98)
plus Nem	41.44(0.77)	8.55(0.54)	489.69(22.05)
Incubation time			
0	*nd*	*nd*	476.44(17.42)
7	*nd*	*nd*	475.72(23.74)
24	39.05(0.42)	5.18(0.11)	366.31(10.60)
47	39.83(1.39)	8.06(0.35)	556.38(23.78)
67	38.11(0.53)	8.83(0.26)	615.25(23.12)
105	43.73(0.86)	10.50(0.31)	386.32(14.00)
*p*(*nematodes*)	*0.003*	*0.002*	*0.178*
*p* (*time*)	*0.000*	*0.000*	*0.000*
*p* (time*nematode)	*0.068*	*0.222*	*0.350*

nd = not determined because the grasses were at seedling stages.

Because there was no statistical interaction between treatments and time, the mean values of all the sampling points are presented for each factor (treatment and time). Values in parentheses indicate the standard error of the mean (n = 4).

## References

[b1] Lu M. . Responses of ecosystem nitrogen cycle to nitrogen addition: a meta-analysis. New Phytol 189, 1040–1050, doi: 10.1111/j.1469-8137.2010.03563.x (2011). 21138438

[b2] Fierer N. . Comparative metagenomic, phylogenetic and physiological analyses of soil microbial communities across nitrogen gradients. Isme J 6, 1007–1017, doi: 10.1038/ismej.2011.159 (2012). 22134642 PMC3329107

[b3] Schimel J. P. & Bennett J. Nitrogen mineralization: Challenges of a changing paradigm. Ecology 85, 591–602, doi: 10.1890/03-8002 (2004).

[b4] Osler G. H. R. & Sommerkorn M. Toward a complete soil C and N cycle: Incorporating the soil fauna. Ecology 88, 1611–1621, doi: 10.1890/06-1357.1 (2007). 17645007

[b5] Bardgett R. D., Denton C. S. & Cook R. Below-ground herbivory promotes soil nutrient transfer and root growth in grassland. Ecology Letters 2, 357–360 (1999).

[b6] Wardle D. A., Yeates G. W., Williamson W. M., Bonner K. I. & Barker G. M. Linking aboveground and belowground communities: the indirect influence of aphid species identity and diversity on a three trophic level soil food web. Oikos 107, 283–294, doi: 10.1111/j.0030-1299.2004.13523.x (2004).

[b7] Griffiths B. S., Christensen S. & Bonkowski M. In The rhizosphere – an ecological perspective (ed Whitbeck J. L. & Cardon Z. G.) (Elsevier, 2004).

[b8] Borgonie G. . Nematoda from the terrestrial deep subsurface of South Africa. Nature 474, 79–82, doi: 10.1038/nature09974 (2011). 21637257

[b9] Ferris H. . Reflections on Plant and Soil Nematode Ecology: Past, Present and Future. J Nematol 44, 115–126 (2012). 23482864 PMC3578467

[b10] Denton C. S., Bardgett R. D., Cook R. & Hobbs P. J. Low amounts of root herbivory positively influence the rhizosphere microbial community in a temperate grassland soil. Soil Biol Biochem 31, 155–165 (1999).

[b11] Yeates G. W., Saggar S., Hedley C. B. & Mercer C. F. Increase in C-14-carbon translocation to the soil microbial biomass when five species of plant-parasitic nematodes infect roots of white clover. Nematology 1, 295–300, doi: 10.1163/156854199508298 (1999).

[b12] Kuzyakov Y. Review: Factors affecting rhizosphere priming effects. J Plant Nutr Soil Sc 165, 382–396, doi: 10.1002/1522-2624(200208)165:4<382::Aid-Jpln382>3.0.Co;2-# (2002).

[b13] Kuzyakov Y., Friedel J. K. & Stahr K. Review of mechanisms and quantification of priming effects. Soil Biol Biochem 32, 1485–1498, doi: 10.1016/S0038-0717(00)00084-5 (2000).

[b14] Ingham R. E., Trofymow J. A., Ingham E. R. & Coleman D. C. Interactions of Bacteria, Fungi, and Their Nematode Grazers - Effects on Nutrient Cycling and Plant-Growth. Ecol Monogr 55, 119–140, doi: 10.2307/1942528 (1985).

[b15] Bonkowski M., Cheng W. X., Griffiths B. S., Alphei G. & Scheu S. Microbial-faunal interactions in the rhizosphere and effects on plant growth. European Journal of Soil Biology 36, 135–147, doi: 10.1016/S1164-5563(00)01059-1 (2000).

[b16] Clarholm M. In Beyond the Biomass (eds Ritz K., Dighton J. & Giller K. E.) 221–230 (Wiley-Sayce, 1994).

[b17] Wardle D. A. & Yeates G. W. The Dual Importance of Competition and Predation as Regulatory Forces in Terrestrial Ecosystems - Evidence from Decomposer Food-Webs. Oecologia 93, 303–306, doi: 10.1007/Bf00317685 (1993). 28313621

[b18] Neher D. A. Role of nematodes in soil health and their use as indicators. Journal of Nematology 33, 161–168 (2001). 19265875 PMC2620512

[b19] Coleman D. C. . An Analysis of Rhizosphere-Saprophage Interactions in Terrestrial Ecosystems. Ecological Bulletins 25, 299–309, doi: http://www.jstor.org/stable/20112592 (1977).

[b20] Chen J. & Ferris H. The effects of nematode grazing on nitrogen mineralization during fungal decomposition of organic matter. Soil Biol Biochem 31, 1265–1279, doi: 10.1016/s0038-0717(99)00042-5 (1999).

[b21] Xiao H. F. . Influence of bacterial-feeding nematodes on nitrification and the ammonia-oxidizing bacteria (AOB) community composition. Applied Soil Ecology 45, 131–137, doi: 10.1016/j.apsoil.2010.03.011 (2010).

[b22] Hunt H. W. . The detrital food web in a short grass prairie. Biology and Fertility of Soils 3, 57–68 (1987).

[b23] Bardgett R. D. & Chan K. F. Experimental evidence that soil fauna enhance nutrient mineralization and plant nutrient uptake in montane grassland ecosystems. Soil Biol Biochem 31, 1007–1014, doi: 10.1016/S0038-0717(99)00014-0 (1999).

[b24] Gebremikael M. T., Buchan D. & De Neve S. Quantifying the influences of free-living nematodes on soil nitrogen and microbial biomass dynamics in bare and planted microcosms. Soil Biol Biochem 70, 131–141, doi: 10.1016/j.soilbio.2013.12.006 (2014).

[b25] Craine J. M., Morrow C. & Fierer N. Microbial nitrogen limitation increases decomposition. Ecology 88, 2105–2113, doi: 10.1890/06-1847.1 (2007). 17824441

[b26] Moorhead D. L. & Sinsabaugh R. L. A theoretical model of litter decay and microbial interaction. Ecol Monogr 76, 151–174, doi: 10.1890/0012-9615(2006)076[0151:Atmold]2.0.Co;2 (2006).

[b27] Huhta V. The role of soil fauna in ecosystems: A historical review. Pedobiologia 50, 489–495, doi: 10.1016/j.pedobi.2006.08.006 (2007).

[b28] Teuben A. Nutrient Availability and Interactions between Soil Arthropods and Microorganisms during Decomposition of Coniferous Litter - a Mesocosm Study. Biology and Fertility of Soils 10, 256–266, doi: 10.1007/Bf00337376 (1991).

[b29] Galloway J., Raghuram N. & Abrol Y. P. Perspective on reactive nitrogen in a global, Asian and Indian context. Curr Sci India 94, 1375–1381 (2008).

[b30] Bockelmann U., Szewzyk U. & Grohmann E. A new enzymatic method for the detachment of particle associated soil bacteria. Journal of Microbiological Methods 55, 201–211, doi: 10.1016/S0167-7012(03)00144-1 (2003). 14500011

[b31] Bonkowski M. & Clarholm M. Stimulation of Plant Growth through Interactions of Bacteria and Protozoa: Testing the Auxiliary Microbial Loop Hypothesis. Acta Protozool 51, 237–247, doi: 10.4467/16890027ap.12.019.0765 (2012).

[b32] Gebremikael M. T., De Waele J., Buchan D., Soboksa G. E. & De Neve S. The effect of varying gamma irradiation doses and soil moisture content on nematodes, the microbial communities and mineral nitrogen. Applied soil ecology 92, 1–13 (2015).

[b33] Buchan D., Moeskops B., Ameloot N., De Neve S. & Sleutel S. Selective sterilisation of undisturbed soil cores by gamma irradiation: Effects on free-living nematodes, microbial community and nitrogen dynamics. Soil Biol Biochem 47, 10–13, doi: 10.1016/j.soilbio.2011.12.014 (2012).

[b34] Treonis A. M. . Effects of organic amendment and tillage on soil microorganisms and microfauna. Applied Soil Ecology 46, 103–110, doi: 10.1016/j.apsoil.2010.06.017 (2010).

[b35] Djigal D., Brauman A., Diop T. A., Chotte J. L. & Villenave C. Influence of bacterial-feeding nematodes (Cephalobidae) on soil microbial communities during maize growth. Soil Biol Biochem 36, 323–331, doi: 10.1016/j.soilbio.2003.10.007 (2004).

[b36] Bardgett R. D., Cook R., Yeates G. W. & Denton C. S. The influence of nematodes on below-ground processes in grassland ecosystems. Plant and Soil 212, 23–33, doi: 10.1023/a:1004642218792 (1999).

[b37] Griffiths B. S., Bonkowski M., Dobson G. & Caul S. Changes in soil microbial community structure in the presence of microbial-feeding nematodes and protozoa. Pedobiologia 43, 297–304 (1999).

[b38] Postma-Blaauw M. B. . Within-trophic group interactions of bacterivorous nematode species and their effects on the bacterial community and nitrogen mineralization. Oecologia 142, 428–439, doi: 10.1007/s00442-004-1741-x (2005). 15526119

[b39] Treseder K. K. & Allen M. F. Direct nitrogen and phosphorus limitation of arbuscular mycorrhizal fungi: a model and field test. New Phytol 155, 507–515, doi: 10.1046/j.1469-8137.2002.00470.x (2002). 33873310

[b40] Mao X. F. . Do bacterial-feeding nematodes stimulate root proliferation through hormonal effects? Soil Biol Biochem 39, 1816–1819, doi: 10.1016/j.soilbio.2007.01.027 (2007).

[b41] Koller R., Rodriguez A., Robin C., Scheu S. & Bonkowski M. Protozoa enhance foraging efficiency of arbuscular mycorrhizal fungi for mineral nitrogen from organic matter in soil to the benefit of host plants. New Phytol 199, 203–211, doi: 10.1111/Nph.12249 (2013). 23534902

[b42] Elsen A., Gervacio D., Swennen R. & De Waele D. AMF-induced biocontrol against plant parasitic nematodes in Musa sp.: a systemic effect. Mycorrhiza 18, 251–256, doi: 10.1007/s00572-008-0173-6 (2008). 18392645

[b43] Buchan D. *The influences of free living nematodes on nitrogen mineralization in agricultural soil* MSc thesis, Gent university (2013).

[b44] Ferris H. & Bongers T. Nematode indicators of organic enrichment. J Nematol 38, 3–12 (2006). 19259424 PMC2586436

[b45] Fu S. L., Ferris H., Brown D. & Plant R. Does the positive feedback effect of nematodes on the biomass and activity of their bacteria prey vary with nematode species and population size? Soil Biol Biochem 37, 1979–1987, doi: 10.1016/j.soilbio.2005.01.018 (2005).

[b46] Trap J., Bonkowski M., Plassard C., Villenave C. & Blanchart E. Ecological importance of soil bacterivores for ecosystem functions. Plant Soil 398, 24, doi: 10.1007/s11104-015-2671-6 (2016).

[b47] Cline G. R., Lindemann W. C. & Quintero R. Dynamics of extractable P during non sterile and sterile incubation of sludge amended soil. Soil Sci 140 (1985).

[b48] De Bolle S., Gebremikael M. T., Maervoet V. & De Neve S. Performance of phosphate-solubilizing bacteria in soil under high phosphorus conditions. Biol Fert Soils 49, 705–714, doi: 10.1007/s00374-012-0759-1 (2013).

[b49] Irshad U., Villenave C., Brauman A. & Plassard C. Grazing by nematodes on rhizosphere bacteria enhances nitrate and phosphorus availability to Pinus pinaster seedlings. Soil Biology & Biochemistry 43, 2121–2126, doi: 10.1016/j.soilbio.2011.06.015 (2011).

[b50] Johansen A., Finlay R. D. & Olsson P. A. Nitrogen metabolism of external hyphae of the arbuscular mycorrhizal fungus Glomus intraradices. New Phytol 133, 705–712, doi: 10.1111/j.1469-8137.1996.tb01939.x (1996).

[b51] Crotty F. V., Blackshaw R. P. & Murray P. J. Tracking the flow of bacterially derived C-13 and N-15 through soil faunal feeding channels. Rapid Commun Mass Sp 25, 1503–1513, doi: 10.1002/rcm.4945 (2011). 21594923

[b52] Ronn R., Vestergard M. & Ekelund F. Interactions between Bacteria, Protozoa and Nematodes in Soil. Acta Protozool 51, 223–235, doi: 10.4467/16890027ap.12.018.0764 (2012).

[b53] Hodge A., Stewart J., Robinson D., Griffiths B. S. & Fitter A. H. Competition between roots and soil micro-organisms for nutrients from nitrogen-rich patches of varying complexity. J Ecol 88, 150–164, doi: 10.1046/j.1365-2745.2000.00434.x (2000).

[b54] Hodge A., Robinson D. & Fitter A. Are microorganisms more effective than plants at competing for nitrogen? Trends in Plant sciences 5, 5 (2000). 10.1016/s1360-1385(00)01656-3 10871903

[b55] Mao X. F., Hu F., Griffiths B. & Li H. X. Bacterial-feeding nematodes enhance root growth of tomato seedlings. Soil Biology & Biochemistry 38, 1615–1622, doi: 10.1016/j.soilbio.2005.12.002 (2006).

[b56] Bonkowski M., Villenave C. & Griffiths B. Rhizosphere fauna: the functional and structural diversity of intimate interactions of soil fauna with plant roots. Plant and Soil 321, 213–233, doi: 10.1007/s11104-009-0013-2 (2009).

[b57] Gusewell S. & Gessner M. O. N: P ratios influence litter decomposition and colonization by fungi and bacteria in microcosms. Functional Ecology 23, 211–219, doi: 10.1111/j.1365-2435.2008.01478.x (2009).

[b58] Tu C., Koenning S. R. & Hu S. Root-parasitic nematodes enhance soil microbial activities and nitrogen mineralization. Microbial Ecology 46, 134–144, doi: 10.1007/s00248-002-1068-2 (2003). 12739076

[b59] Six J., Frey S. D., Thiet R. K. & Batten K. M. Bacterial and fungal contributions to carbon sequestration in agroecosystems. Soil Sci Soc Am J 70, 555–569, doi: 10.2136/sssaj2004.0347 (2006).

[b60] Haase S., Ruess L., Neumann G., Marhan S. & Kandeler E. Low-level herbivory by root-knot nematodes (Meloidogyne incognita) modifies root hair morphology and rhizodeposition in host plants (Hordeum vulgare). Plant and Soil 301, 151–164, doi: 10.1007/s11104-007-9431-1 (2007).

[b61] Karssen G. & Moens M. In Plant nematology. (eds Perry R. N. & Moens M.) 59–88. (CABI, 2006).

[b62] Jackson M. L. Soil chemical analysis. 326–336 (Prentice Hall, 1958).

[b63] Buchan D., Gebremikael M. T., Ameloot N., Sleutel S. & De Neve S. The effect of free-living nematodes on nitrogen mineralisation in undisturbed and disturbed soil cores. Soil Biology & Biochemistry 60, 142–155 (2013).

[b64] Murphy J. & Riley J. P. A Modified Single Solution Method for Determination of Phosphate in Natural Waters. Anal Chim Acta 26, 31-& (1962).

[b65] Hogue E., Wilcox G. E. & Cantliff. Dj. Effect of Soil Phosphorous Levels on Phosphate Fractions in Tomato Leaves. J Am Soc Hortic Sci 95, 174-& (1970).

