# Supplementary material for: Nematodes enhance plant growth and nutrient uptake under C and N-rich conditions
Source: Sci Rep. 2016 Sep 8;6:32862. doi: 10.1038/srep32862 (PMC5015107; doi:10.1038/srep32862)
Supplement: Supplementary Information [file srep32862-s1.pdf]

Supporting information

Nematodes enhance plant growth and nutrient uptake under C and N-rich conditions

Mesfin T. Gebremikael[a]*, Hanne Steel[b], David Buchan[a], Wim Bert[b], and Stefaan De Neve[a]

Affiliations:

[a]Department of Soil Management, University of Ghent, Ghent, Belgium

[b]Department of Biology, Nematology Research Unit, University of Ghent, Ghent, Belgium

*Corresponding author

mesfintsegaye.gebremikael@ugent.be

S1. Materials and methods

Nematode extraction, identification and analysis of community indices

Throughout the incubation, all free-living nematodes were extracted from 75 g moist soil of +Nem treatments using an automated zonal centrifuge [1]. Nematodes were extracted from the CTR samples only at the beginning of the experiment to check whether reinoculation was successful both in terms of abundance and diversity. After extraction, the nematode suspension was poured onto a sieve and covered with a filter paper for 72 hours so that only viable nematodes migrated towards the water and were collected at the bottom of the plate. All viable nematodes that passed through the filter were then concentrated and counted using a dissecting microscope (40x). Mass fixation was carried out by adding hot 4% formaldehyde (65°C) at a ratio of 1:2 (5 ml nematode suspension and 10 ml formaldehyde) followed by immediately cooled in cold water according to the procedure by van Bezooijen [2]. After six weeks of storage at room temperature, the formaldehyde was tapped off carefully until 1 ml of the nematode suspension remained, into which glycerin was added at a 1:1 ratio. Slides were prepared for each sample, and at least 150 individual nematodes were identified from each slide to family or where possible to genus level based on Bongers [3] and then assigned to the corresponding functional groups according to Yeates, et al. [4]. However, *Filenchus* was considered as fungivorous as suggested by different authors [5-7] and the remaining genera of the Tylenchidae family considered as root feeders. Nematode identification was carried out at the beginning (day 7), middle (day 47) and end of the incubation (day 105).

Maturity and community indices (channel, structural and enrichment indices) of nematodes were calculated according to Bongers [8] and Ferris, et al. [9]. Each nematode taxa identified in the sample was first classified into a colonizers-persisters (cp) scale (from cp1=colonizers to cp5=persisters) based on their natural attributes such as reproduction rate according to Bongers [8]. Nematode taxa that multiply fast in response to nutrient inputs are considered to be colonizers, and taxa that are long-lived and require stable conditions to establish are considered to be persisters. The maturity index (MI) is the weighted mean frequency of all free-living nematodes excluding plant parasitic nematodes. MI2-5 is a maturity index excluding enrichment opportunist (cp1) nematodes. The plant parasitic index (PPI) is the weighted mean frequency of all plant-parasitic nematodes. Nematodes classified as cp2 were considered basal (b) to both enrichment (EI) and structure indices (SI) [9]. Bacterial-feeding cp1 and fungal-feeding cp2 were considered as indicators of enrichment (e). Nematodes of all feeding habits classified in cp3-5 are indicators of structure (s).

Microbial Biomass C and PLFA

Microbial biomass C was determined by fumigation and extraction following the procedure by Vance et al. (1987). Changes in the structure of the microbial community were determined using phospholipid fatty acid (PLFA) extraction according to the procedure explained in detail in Buchan et al. (2013). Briefly, 4 g of freeze-dried soil from each sample was sieved (2 mm) in order to homogenize and remove root fragments and stones. Phospholipids were extracted from this freeze-dried soil and transformed into methyl esters (FAMEs). Finally, individual FAMEs were identified and quantified by Gas Chromatography-Mass Spectrometry (GC-MS) on a Thermo Focus GC combined with a Thermo DSQ quadrupole MS (Interscience BVBA, Louvain-la-Neuve, Belgium) in the electron ionization mode.

The sums of marker fatty acid concentrations for selected microbial groups were calculated as follows. For Gram-positive bacteria the sum of i15:0, a15:0, i16:0, a16:0, i17:0 and a17:0; for Gram-negative bacteria cy17:0, cy19:0, C16:1ω7, C16:1ω9; for the actinomycetes the sum of 10-methyl branched saturated fatty acids (Frostegård et al., 1996; Buyer and Sasser 2012). For the total bacterial community, in addition to Gram-positive and Gram-negative bacteria, the fatty acids 15:0, 17:0 were also included [10]. For saprotrophic fungi the marker PLFAs 18:2ω6c and 18:1ω9, and for arbuscular mycorrhizal fungi (AMF) 16:1ω5c were considered [11]. The fungal: bacterial ratio was calculated as saprotrophic fungal marker fatty acids divided by the sum of total bacterial marker fatty acids. PLFAs 20:3ω6 and 20:4 were considered as a biomarker for protozoa (Ringelberg et al., 1997; Buyer and Sasser 2012).

S2. Results

Table S1. Mean nematode abundances (ind. g^{-1} soil) and SE of the mean (n=3) in unirradiated fresh soil (CTR) and irradiated and reinoculated soil (+Nem) at the start of the incubation experiment as shown in Gebremikael, et al. [12]. The same small letter 'a' refers to no significant differences between CTR and +Nem.

Family/Genus	Feeding groups	Mean abundance (individuals g^{-1} soil) ± SE	
		CTR	Reinoculated (+Nem)
Coslenchus	Plant and root	0.48±0.01	1.56±0.23
Dolichodoridae	Plant and root	0.24±0.06	0.70±0.09
Heteroderinae	Plant and root	1.95±0.19	1.28±0.21
Tylenchidae	Plant and root	2.08±0.11	2.09±0.73
Paratylenchus	Plant and root	0.59±0.08	1.47±0.41
Meloidogyne	Plant and root	0.83±0.02	0.33±0.18
Pratylenchus	Plant and root	0.18±0.04	0.18±0.09
Psilenchus	Plant and root	0.12±0.00	0.22±0.11
∑herbivores		**6.47±0.08a**	**7.84±0.5a**
Filenchus	Fungivorous	0.18±0.04	0.40±0.03
Aphelenchoides	Fungivorous	0.11±0.09	0.24±0.11
Aphelenchus	Fungivorous	0.34±0.28	0.49±0.07
∑fungivores		**0.64±0.51a**	**1.18±0.25a**
Panagrolaimidae	Bacterivorous	0.06±0.05	0.37±0.31
Plectus	Bacterivorous	0.06±0.05	0.08±0.08
Cephalobidae	Bacterivorous	4.68±0.21	3.49±0.38
Pristionchus	Bacterivorous	0.11±0.09	0.49±0.07
Rhabditidae	Bacterivorous	5.93±0.32	4.30±0.48
∑bacterivores		**10.84±0.88a**	**8.69±0.94a**
Seinura	Predatory	0.00±0.00	0.04±0.04
Steinernema	Bacterivorous and Entomopathogenic	0.00±0.00	0.04±0.04
Nygolaimidae	Carnivorous/predatory	0.08±0.02	0.05±0.05
Dorylaimidae	Omnivorous	0.36±0.20	0.17±0.11
∑predators/omnivores		**0.36±0.25a**	**0.30±0.18a**

**Total abundance**	**18.31±1.00a**	**18.02±1.40a**

Table S2. Comparison of nematode indices between unirradiated CTR and +Nem at the beginning of the incubation and evolution of nematode indices over time in +Nem treatments. The data corresponds to mean values (n=3) and standard errors of the mean. Significant differences were found only at the end of the incubation period (day 105) as indicated by different small letters.

	Days	PPI	MI	MI 2-5	EI (%)	SI (%)	CI (%)
CTR	7	2.57 ± 0.01a	1.55 ± 0.04a	2.14 ±0.08a	82.39 ±1.46a	20.99±11.51a	2.34±1.46a
+Nem	7	2.52 ± 0.03a	1.55± 0.07a	2.11 ±0.06a	82.01 ±1.91a	18.35±9.67a	5.81±1.91a
+Nem	47	2.51 ± 0.06a	1.73 ±0.05a	2.10 ±0.03a	69.21 ±2.27a	16.61±4.20a	11.71±2.27a
+Nem	105	2.55 ± 0.03a	1.94 ± 0.02b	2.01 ±0.01a	39.13 ±9.19b	2.01±1.00b	56.72±9.19b

***PPI**= plant parasitic index, **MI**= maturity index, **MI2-5**= maturity index without early colonizers (cp1) nematodes, **EI**= enrichment index, **SI**= structural index, **CI**= channel index [9].*

Table S3: Abundances of PLFA biomarkers (mean+SE) for major microbial groups in the unirradiated CTR and +Nem treatments after seven days of incubation. The F and p values are after one-way analysis of variance.

	**Total PLFA**	**Gram-positive bacteria**	**Gram-negative bacteria**	**Actinomycetes**	**Saprophytic fungi**	**Arbuscular mycorrhizal fungi**	**Protozoa**
CTR	54.9+7.65	11.69±1.57	18.62±2.18	4.42±0.51	4.4±0.83	2.02±0.24	0.27±0.04
+Nem	46.32±0.57	8.48±0.01	15.34±0.31	2.19±0.015	4.41±0.08	1.51±0.01	0.21±0.05
F value	1.26	4.17	2.21	19.48	0.12	4.49	1.76
p value	*0.38*	*0.18*	*0.27*	*0.05*	*0.76*	*0.17*	*0.32*

Table S4. Two-way ANOVA output (p and F values) of the two factors and their interaction (time and treatment). The symbol asterisk (*) indicates significant interactions which are also displayed in the corresponding figures in the main text.

Factors/ Plant and soil parameters	time		treatment		Time*treatment	
	F value	p	F value	p	F value	p
N uptake (mg pot^{-1})	12.54	0.000	13.12	0.001	2.70	0.068
P uptake (mg pot^{-1})	97.55	0.000	13.11	0.001	1.57	0.222
Total dry biomass (mg pot^{-1})	633.35	0.000	24.05	0.000	5.37	0.006*
Total min N (mg kg^{-1} soil)	680.31	0.000	1.49	0.23	0.49	0.780
Plant N + Soil N (mg kg^{-1} soil)	70.74	0.000	3.93	0.055	2.92	0.026*
Water extractable P (mg kg^{-1} soil)	95.96	0.000	0.03	0.864	2.37	0.058
Plant P + Soil P (mg kg^{-1} soil)	11.88	0.001	3.92	0.000	3.65	0.009*
C mic (mg kg^{-1} soil)	25.13	0.000	1.88	0.178	1.16	0.350
NH4+-N (mg kg-1 soil)	811.5	0.000	0.87	0.362	0.19	0.643
NO_3^--N (mg kg^{-1} soil)	21.6	0.000	3.56	0.071	1.29	0.130
Total mineral N (mg kg^{-1} soil)	680.3	0.000	1.49	0.231	0.49	0.783
Soil P (mg kg^{-1} soil)	82.1	0.000	0.025	0.870	2.04	0.065
Total PLFA (nmol µg^{-1} soil)	2.46	0.051	8.41	0.006	4.64	0.002*
Gram +ve bacteria (nmol µg^{-1} soil)	6.82	0.000	0.01	0.962	2.13	0.081
Gram -ve bacteria (nmol µg^{-1} soil)	1.64	0.174	0.95	0.337	1.14	0.362
Actinomycetes (nmol µg^{-1} soil)	2.19	0.077	0.78	0.784	1.17	0.341
Saprophytic fungi (nmol µg^{-1} soil)	22.9	0.000	44.3	0.000	20.5	0.001*
AMF (nmol µg^{-1} soil)	8.52	0.000	7.94	0.008	9.53	0.000*
Protozoa (nmol µg^{-1} soil)	14.09	0.000	16.43	0.000	13.88	0.000*

References

1 Hendrickx, G. An automatic apparatus for extracting free-living nematode stages from soil *Nematologica* **41** 308 (1995).

2 van Bezooijen, J. *Methods and Techniques for Nematology*. (2006).

3 Bongers, T. *De Nematoden van Nederland. Utrecht: Stichting Uitgeverij Koninklijke Nederlandse Natuurhistorische Vereniging*. (Koninklijke Nederlandse Natuurhistotische Vereniging, 1994).

4 Yeates, G. W., Bongers, T., Degoede, R. G. M., Freckman, D. W. & Georgieva, S. S. Feeding-Habits in Soil Nematode Families and Genera - an Outline for Soil Ecologists. *Journal of Nematology* **25**, 315-331 (1993).

5 Buchan, D., Moeskops, B., Ameloot, N., De Neve, S. & Sleutel, S. Selective sterilisation of undisturbed soil cores by gamma irradiation: Effects on free-living nematodes, microbial community and nitrogen dynamics. *Soil Biol Biochem* **47**, 10-13, doi:DOI 10.1016/j.soilbio.2011.12.014 (2012).

6 Okada, H., Tsukiboshi, T. & Kadota, I. Mycetophagy in Filenchus misellus (Andrassy, 1958) Lownsbery & Lownsbery, 1985 (Nematoda : Tylenchidae), with notes on its morphology. *Nematology* **4**, 795-801, doi:10.1163/156854102760402586 (2002).

7 Christensen, S., Alphei, J., Vestergard, M. & Vestergaard, P. Nematode migration and nutrient diffusion between vetch and barley material in soil. *Soil Biol Biochem* **39**, 1410-1417, doi:DOI 10.1016/j.soilbio.2006.11.026 (2007).

8 Bongers, T. The Maturity Index, the evolution of nematode life history traits, adaptive radiation and cp-scaling. *Plant and Soil* **212**, 13-22, doi:Doi 10.1023/A:1004571900425 (1999).

9 Ferris, H., Bongers, T. & de Goede, R. G. M. A framework for soil food web diagnostics: extension of the nematode faunal analysis concept. *Applied Soil Ecology* **18**, 13-29, doi:Doi 10.1016/S0929-1393(01)00152-4 (2001).

10 Moeskops, B. *et al.* Soil microbial communities and activities under intensive organic and conventional vegetable farming in West Java, Indonesia. *Appl Soil Ecol* **45**, 112-120, doi:DOI 10.1016/j.apsoil.2010.03.005 (2010).

11 Joergensen, R. & Wichern, F. Quantitative assessment of the fungal contribution to microbial tissue in soil. *Soil Biology and Biochemistry* **40**, 2977-2991, doi:10.1016/j.soilbio.2008.08.017 (2008).

12 Gebremikael, M. T. *et al.* Quantifying the Contribution of Entire Free-Living Nematode Communities to Carbon Mineralization under Contrasting C and N Availability. *Plos One* **10**, doi:ARTN e013624410.1371/journal.pone.0136244 (2015).
